# Efficacy and safety of enzyme-replacement-therapy with agalsidase alfa in 36 treatment-naïve Fabry disease patients

**DOI:** 10.1186/s40360-017-0152-7

**Published:** 2017-06-07

**Authors:** Kazuya Tsuboi, Hiroshi Yamamoto

**Affiliations:** LSD Center, Nagoya Central Hospital, 3-7-7 Taiko, Nakamura-ku, Nagoya, 453-0801 Japan

**Keywords:** Fabry disease, Enzyme replacement therapy (ERT), Agalsidase Alfa, Globotriaosylceramide (Gb3), Globotriaosylsphingosine (lyso-Gb3)

## Abstract

**Background:**

Fabry disease (FD) is an X-linked lysosomal storage disorder resulting from the α-galactosidase A gene mutations. Enzyme-replacement-therapy (ERT) products for FD currently used include agalsidase alfa and agalsidase beta. There are many reports on efficacy and safety of ERT. However, most of the previous studies are done as a retrospective medical records analysis.

**Methods:**

The Japan Fabry Research - 002 (JFR-002) was a prospective observational clinical study of 36 ERT-naïve FD patients (14 men and 22 women) at baseline (BL) and after initiation of ERT with agalsidase alfa 0.2 mg/kg every two weeks, a median period 62.5 months. The parameters measured included globotriaosylceramide (Gb3), globotriaosylsphingosine (Lyso-Gb3), left ventricular mass index (LVMI), brain natriuretic peptide (BNP), high-sensitivity troponin I (hs-Trop I), estimated glomerular filtration rate (eGFR), and anti-agalsidase alfa IgG antibody formation.

**Results:**

All parameters remained steady during ERT treatment period. BNP levels in 14 patients whose BL levels were within the normal range (<19.5 pg/mL) remained within the same range, while 22 patients whose BL levels were abnormally high (≥19.5 pg/mL) gradually showed decreased levels after start of ERT. Gb3 and Lyso-Gb3 levels remarkably decreased after the initiation of ERT and remained low.

**Conclusion:**

The JFR-002 suggests that agalsidase alfa is effective in maintaining organ function in FD patients, and that the incidence of infusion reactions related to the treatment with agalsidase alfa is low, indicating the good tolerability to this ERT.

**Trial registration:**

The JFR-002 was retrospectively registered at Japan Medical Association Center for Clinical Trials (Registration number: JMA-IIA00291) on May 19th, 2017.

## Background

Fabry disease (FD, OMIM number #301500) is an inborn error of metabolism caused by the reduced or deficient α-galactosidase A (α-Gal) activity in cells. This leads to the progressive accumulation of glycosphingolipids, including globotriaosylceramide (Gb3), in many tissues and body fluids, such as in the walls of blood vessels and vascular endothelial cells throughout the body, as well as in parts of the nervous system. The gene locus for this enzyme is located on the X chromosome (Xq21.33-q22) and the mode of inheritance is X-linked. Hemizygous men experience pain in the extremities, angiokeratoma, hypohidrosis, corneal opacity, and vascular disorders of the heart, kidneys, and brain, while heterozygous women develop various symptoms ranging from asymptomatic to severe [1–3].

Enzyme-replacement-therapy (ERT) products currently used for FD include agalsidase alfa and agalsidase beta [2–4]. There were numerous reports on efficacy and safety of ERT with agalsidase alfa or agalsidase beta [5–9]. However, most of the previous studies were done as a retrospective, medical records analysis, so it would be difficult to control and remove inter-institutional bias. It would also be an issue to keep accuracy of clinical records.

The Japan Fabry Research - 002 (JFR-002) was a prospective, observational study for 5 years to evaluate efficacy and safety of ERT with agalsidase alfa in patients with FD. All treatments and clinical evaluations to the patients who were enrolled in the study were also conducted at only one institute. Various parameters including cardiac function, renal function, blood and urine tests, pain score and quality of life (QoL) score as well as Gb3 and globotriaosylsphingosine (Lyso-Gb3) as a biomarker were regularly measured, and efficacy and safety of ERT with agalsidase alfa of 0.2 mg/kg every two weeks were evaluated.

## Methods

### Patients

The JFR-002 was a prospective observational study, where patients, who had been initiated on ERT with agalsidase alfa as part of the individual treatment decision between physician and patient, were followed up routinely. ERT treatment-naïve FD patients were prospectively enrolled into this observational clinical trial based on the following inclusion and exclusion of criteria. The study period was to cover 5 years of ERT follow-up.

Inclusion criteria:

1. ERT treatment-naïve patients with FD
2. Patients receiving treatment with agalsidase alfa 0.2 mg/kg every 2 weeks

Exclusion criteria:

1. Patients with E66Q amino acid substitution [10].

The study was conducted in accordance with the Declaration of Helsinki and applicable local laws and regulations. Patients provided written informed consent before inclusion into the study.

### Hematological and blood chemistry tests

Patients’ pre-ERT parameter levels were set as BL and every month regularly assessed with hematological tests, including red blood cell (RBC) count, hemoglobin (Hb), hematocrit (Ht), white blood cell (WBC) count, and platelet (Plt) count. Similarly, blood chemistry tests were carried out to measure total protein (TP), albumin (Alb), total bilirubin (T-Bil), alkaline phosphatase (ALP), aspartate transaminase (AST), alanine aminotransferase (ALT), gamma-glutamyl transferase (γ-GTP), total cholesterol (T-Cho), high-density lipoprotein (HDL), triglyceride (TG), low-density lipoprotein (LDL), lactase dehydrogenase (LDH), creatinine kinase (CK), blood urea nitorogen (BUN), uric acid (UA), creatinine (Cre), estimated glomerular filtration rate (eGFR), sodium (Na), chlorine (Cl), potassium (K), C-reactive protein (CRP), free triiodothyronine (FT3), free thyroxine (FT4), and thyroid-stimulating hormone (TSH).

### Cardiac examination

For assessment of cardiac function, interventricular septal thickness in diastole (IVSd), left ventricular posterior wall thickness in diastole (LVpwd), left ventricular mass index (LVMI), and left ventricular ejection fraction (EF) were measured every 6-months using ultrasonic cardiography (UCG), chest X-ray, and electrocardiography (ECG). Also, brain natriuretic peptide (BNP), and high-sensitivity troponin I (hs-Trop I) were measured every month.

### Renal examination

For assessment of renal function, eGFR, urine protein/creatinine ratio (UP/Cr ratio), beta2-microglobulin (β2-MG), cystatin C, and N-acetyl-beta-D-glucosaminidase (NAG) were measured every month.

### Pain score and QoL assessment

In addition to plasma Gb3 and Lyso-Gb3 [11] used as biomarkers of FD, pain score and QoL were assessed once or twice a year, using brief pain inventory (BPI) [12], Euro QoL- 5 dimension (EQ-5D) [13] and EQ-visual analogue scale (EQ-VAS) [13],respectively, every six months.

### Assessment of adverse events

All patients underwent measurement of vital signs (blood pressure (BP), pulse rate, and body temperature), and were examined for the incidence of infusion related reactions (rashes, chills, nasal discharge, etc.) both immediately before and after each treatment with agalsidase alfa. In addition, the development of anti-agalsidase alfa IgG antibodies was periodically evaluated, every six or twelve months, after the start of ERT. These results were then analyzed to examine the efficacy and safety of the ERT for FD.

### Data analysis

Data are presented as mean ± SD. Paired *t*-test was utilized to compare before and after treatment with ERT. *P* <0.05 was considered statistically significant.

## Results

Thirty-six ERT treatment-naïve FD patients (14 male patients, mean age: 26.6 years old; 22 female patients, mean age: 45.4 years old) (Table 1) were prospectively enrolled into this clinical observational trial based on the inclusion and exclusion of criteria. The median period of treatment observation was 62.5 months (M) (range: 8 M - 84 M).

**Table 1:** Patients baseline characteristics

	Male				Female			
Number of patients	14				22			
Enzyme activity (Agal U)^a^	3.9	±	1.2		15.2	±	6.6	
Onset (y.o.)^a^	7.7	±	7.7		13.5	±	10.7	
Age (y.o.)^a^	26.6	±	10.4		45.4	±	16.4	
	*N*	(	%	)	*N*	(	%	)
Pain attack	11	(	78.6	)	6	(	27.3	)
Angiokeratoma	7	(	50	)	4	(	18.2	)
Hypohidrosis	10	(	71.4	)	4	(	18.2	)
Depression	1	(	7.1	)	3	(	13.6	)
Stroke	2	(	14.3	)	2	(	9.1	)
Corneal opacity	10	(	71.4	)	16	(	72.7	)
Tinnitus	6	(	42.9	)	4	(	18.2	)
Dizziness	0	(	0	)	5	(	22.7	)
Diarrhea	7	(	50	)	8	(	36.4	)
Hypertension	1	(	7.1	)	1	(	4.5	)
Angina	3	(	21.4	)	3	(	13.6	)
LVH^b^	1	(	7.1	)	9	(	40.9	)
Proteinuria	5	(	35.7	)	4	(	18.2	)
Renal failure	0	(	0	)	2	(	9.1	)

^a^mean ± SD, ^b^ *LVH* Left ventricular hypertrophy

### Hematological findings

The values of all hematological parameters remained steady from BL to after follow-up. All hematological parameters did not reveal a statistical significant after treatment with agalsidase alfa due to limitation of the number of patients. In addition, no platelet-count decrease or other adverse events related to the treatment with agalsidase alfa were observed (Table 2).

**Table 2:** Vital signs and laboratory results at baseline and after follow-up

	Baseline			After follow up			Frequency
	Mean	±	SD	Mean	±	SD	
Vital signs (*n* = 36)							
Systolic BP (mmHg)	123.0	±	14.9	125.9	±	14.4	every 2 weeks
Diastolic BP (mmHg)	65.4	±	10.9	67.8	±	11.2	every 2 weeks
Pulse (/min)	69.1	±	15.3	68.7	±	13.0	every 2 weeks
Hematological test (*n* = 36)							
WBC (10^2^/mL)	59.7	±	18.2	60.2	±	15.0	once a month
RBC (10^4^/mL)	445.0	±	34.2	446.5	±	40.4	once a month
Hg (g/dL)	13.2	±	1.2	13.1	±	1.5	once a month
Ht (%)	39.6	±	3.2	39.2	±	3.7	once a month
Plt (10^4^/mL)	23.0	±	6.0	23.4	±	6.7	once a month
Blood chemistry test (*n* = 36)							
TP (g/dL)	7.1	±	0.4	7.1	±	0.4	once a month
Alb (g/dL)	4.4	±	0.2	4.3	±	0.2	once a month
T-Bil (mg/dL)	0.5	±	0.2	0.5	±	0.3	once a month
ALP (U/L)	261.4	±	155.1	239.8	±	72.2	once a month
AST (U/L)	22.9	±	10.4	24.5	±	8.2	once a month
ALT (U/L)	18.0	±	17.8	19.7	±	12.0	once a month
y-GTP (U/L)	29.9	±	25.5	37.2	±	29.5	once a month
T-Cho (mg/dL)	197.3	±	39.0	198.4	±	37.7	once a month
HDL (mg/dL)	72.7	±	14.2	74.9	±	14.2	once a month
TG (mg/dL)	100.4	±	65.4	108.5	±	48.7	once a month
LDL (mg/dL)	111.4	±	31.6	116.0	±	31.5	once a month
LDH (U/L)	208.8	±	53.0	233.0	±	67.6	once a month
CK (U/L)	103.4	±	49.0	107.9	±	50.3	once a month
BUN (mg/dL)	12.3	±	3.6	14.8	±	12.3	once a month
UA (mg/dL)	4.7	±	1.3	4.8	±	0.9	once a month
Cre (mg/dL)	0.71	±	0.13	0.92	±	1.19	once a month
eGFR (mL/min/1.73 m^2^)	83.5	±	28.6	82.6	±	29.4	once a month
Na (mEq/L)	141.0	±	2.0	140.8	±	2.0	once a month
Cl (mEq/L)	105.0	±	2.3	105.7	±	1.5	once a month
K (mEq/L)	4.2	±	0.3	4.4	±	0.4	once a month
CRP (mg/dL)	0.30	±	1.17	0.42	±	1.54	once a month
hs-Trop I (pg/mL)	56.9	±	116.8	58.0	±	89.6	once a month
BNP (mg/dL)	86.3	±	149.4	62.2	±	72.8	once a month
β2-MG (mg/L)	1.37	±	0.35	1.78	±	1.98	once a month
Cystatin C (mg/L)	0.67	±	0.11	0.94	±	0.90	once a month
FT3 (pg/mL)	2.79	±	0.38	2.94	±	0.35	every 6 months
FT4 (ng/dL)	1.07	±	0.14	1.00	±	0.15	every 6 months
TSH (mg/dL)	1.64	±	0.90	1.60	±	0.82	every 6 months
Urinalysis (*n* = 36)							
UP/Cr ratio	0.19	±	0.27	0.39	±	0.72	once a month
NAG (U/L)	4.5	±	3.0	3.8	±	2.4	once a month
anti-αGal Ab (*n* = 36)	negative (*n* = 36)			negative (*n* = 35)			every 6 or 12 months
	positive (*n* = 0)			positive (*n* = 1)			

### Blood chemistry results

The values of all blood chemistry parameters remained steady and revealed no statistical significant after treatment. No drug-induced liver disorder or other adverse events related to the treatment with agalsidase alfa were observed (Table 2).

### Cardiac dysfunction

**Fig. 1:**
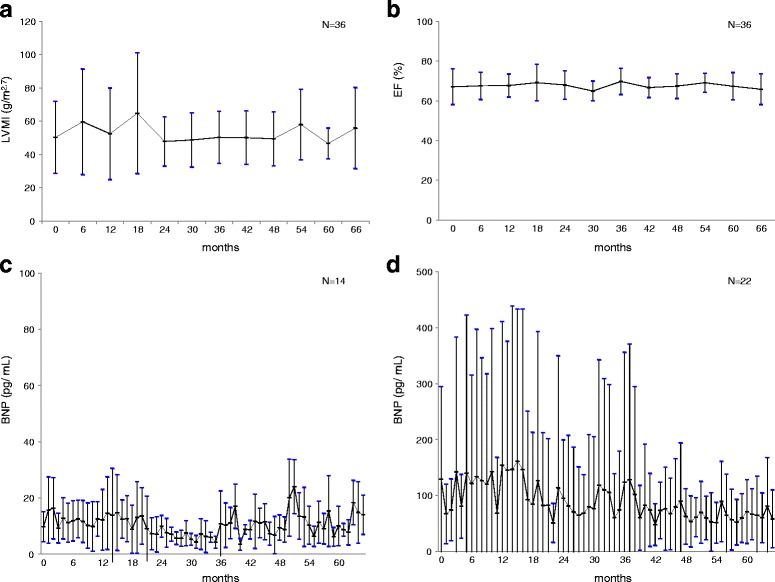
LVMI and BNP levels. For the assessment of cardiac function, LVMI (**a**), and EF (**b**) were measured. LVMI and EF remained steady (mean ± SD). Patients were classified into two groups according to the BNP levels. The BNP levels in grade 1 patients (*n* = 14, <19.5 pg/mL) (**c**) remained within the normal range, and those in grade 2 patients (*n* = 22, ≥19.5 pg/mL) (**d**) gradually decreased throughout ERT period (mean ± SD)

All measurements of IVSd, LVpwd, LVMI, and EF remained stable (IVSd; from 10.5 mm (BL) to 9.9 mm (60 M), LVpwd; from 10.5 mm (BL) to 9.9 mm (60 M), LVMI; from 50.3 g/m^2.7^ (BL) to 46.6 g/m^2.7^ (60 M), EF; from 67.1% (BL) to 67.4%(60 M)) (Fig. 1a and b). The BNP levels in 14 patients, classified as grade 1, whose BL levels were within the normal range (<19.5 pg/mL) remained within the same range from 9.8 pg/mL (BL) to 10.0 pg/mL (60 M), while 22 patients, classified as grade 2, whose BL values were abnormally high (≥19.5 pg/mL) showed a gradual decrease from 129.5 pg/mL (BL) to 71.4 pg/mL (60 M), during the ERT period (Fig. 1c and d). The hs-Trop I levels in 20 patients, classified as grade 1, whose BL levels were within the normal range (<26.2 pg/mL) remained within the same range. On the other hand, among 16 patients, classified as grade 2, whose BL levels were abnormally high (≥26.2 pg/mL), hs-Trop I levels increased in two patients from 10.2 pg/mL (BL) to 59.6 pg/mL (60 M) but remained generally stable in the remaining 34 patients (20 with grade 1 and 14 with grade 2). In the two patients (one woman was grade 1 and another woman was grade 2) with increased hs-Trop I levels, marked myocardial hypertrophy was noted, and the myocardial disorder might have raised the hs-Trop I levels. The other organs such as kidneys and cerebral blood vessels showed no pathologic findings. Careful follow-up observation will be required.

### Renal dysfunction

**Fig. 2:**
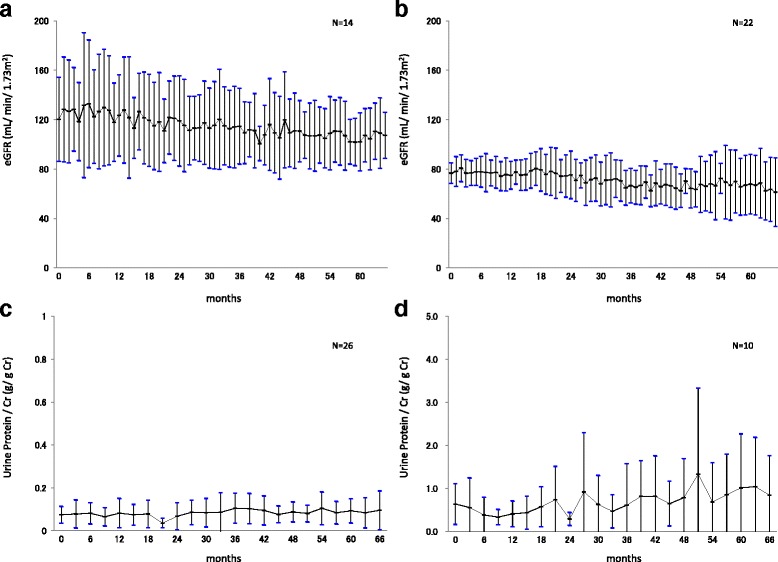
eGFR and UP/Cr ratios. Patients classified according to the eGFR values specified in the CKD guideline: 14 patients in G1 (≥90 mL/min/1.73 m^2^) (**a**), 22 patients in G2 (60–89 mL/min/1.73 m^2^), and G3 (30–59 mL/min/1.73 m^2^) (**b**). Patients classified according to the value of UP/Cr ratios specified in the CKD guideline: 26 patients in A1 (<0.15 g/gCr) (**c**), 10 patients in A2 (0.15–0.49 g/gCr), and A3 (≥0.50 g/gCr) (mean ± SD) (**d**)

The evaluation of renal function was performed according to the classification described in the guidelines for chronic kidney disease (CKD) [14]. The eGFR values in 14 patients classified in G1 (≥90 mL/min/1.73 m^2^) remained within the normal range from 120.4 mL/min/1.73 m^2^ (BL) to 102.1 mL/min/1.73 m^2^ (60 M). Among 17 patients classified in G2 (60–89 mL/min/1.73 m^2^) and 5 patients classified in G3 (30–59 mL/min/1.73 m^2^), the eGFR value in one patient showed a trend to worsen from 80.5 mL/min/1.73 m^2^ to 22.3 mL/min/1.73 m^2^ (60 M), while those of the other 21 patients remained stable from 76.7 mL/min/1.73 m^2^ (BL) to 67.8 mL/min/1.73 m^2^ (60 M) (Fig. 2a and b). The values of UP/Cr ratios in 26 patients classified in A1 (<0.15 g/gCr) remained within the normal range from 0.07 g/g Cr (BL) to 0.09 g/g Cr (60 M). Among four patients classified in A2 (0.15–0.49 g/gCr) and six patients classified in A3 (≥0.50 g/gCr), the value of UP/Cr ratio in one patient showed a trend to worsen, while those of the other nine patients remained stable from 0.64 g/g Cr (BL) to 1.01 g/g Cr (60 M) (Fig. 2c and d).

### Pain scores

**Fig. 3:**
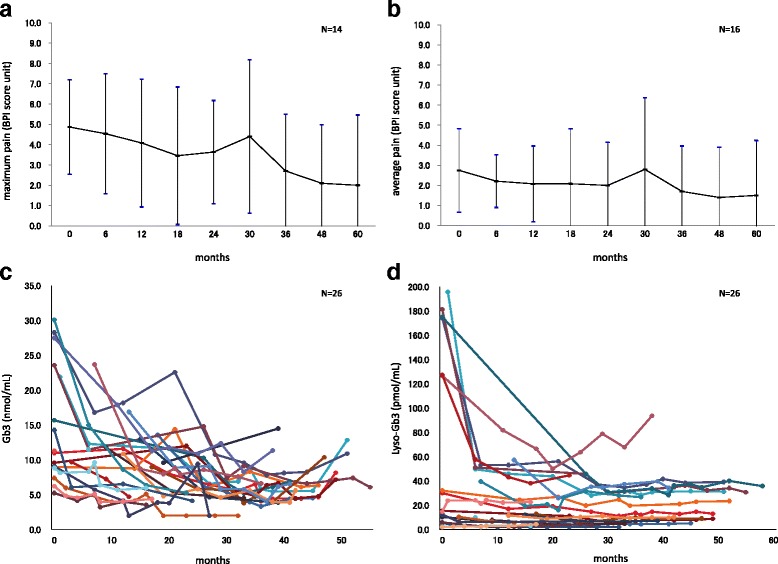
BPI scores and Lyso-Gb3 levels. Using the BPI scores, pain in 14 patients who had a maximum pain of ≥3.0 units at BL improved (**a**) and pain in 16 patients who had an average pain of ≥1.0 units at BL also improved during the ERT period (**b**). The plasma Gb3 (**c**) and Lyso-Gb3 (**d**) levels were considered as the biomarker of FD and they were markedly decreased particularly in male patients

The effects of the ERT on pain were evaluated using BPI scores [12] ranging from zero units (no pain) to 10 units (worst possible pain). The pain scores in 14 patients (6 men, 8 women) who had a maximum pain of ≥3.0 units at BL, improved during the ERT period from 4.87 units (BL) to 2.00 units (60 M), while 2 other patients who had <3.0 units at BL remained stable. The pain scores in 16 patients (8 men and 8 women) who had an average pain of ≥1.0 units at BL, improved during the ERT period from 2.75 units (BL) to 1.50 units (60 M), and while 20 patients who had <1.0 units at BL remained stable (Fig. 3a and b). Paired *t*-test was utilized to compare before and after treatment with ERT. The Pain score did not reveal a statistical significant after treatment with agalsidase alfa due to limitation of the number of patients, but showed a tendency to improvement.

### QoL scores

The effects of ERT on QoL were evaluated using EQ-5D [13] in which the QoL assessments were converted into utility scores (1 representing full health and 0 representing death). The utility scores remained stable, from 0.865 (BL) to 0.7982 (60 M). The EQ-VAS scores [13] were slightly improved from 74.21 (BL) to 79.78 (60 M), because of the pain reduction (Fig. 3a and b).

### Plasma Gb3 and lyso-Gb3 levels

Both plasma Gb3 and Lyso-Gb3 levels were measured in 26 patients. The mean plasma Gb3 level was 14.48 nmol/mL (control < 4.6 ± 2.0 nmol/mL) at BL, 18.82 nmol/mL in male patients (*n* = 12) and 9.53 nmol/mL in female patients (*n* = 14). The average Lyso-Gb3 level was 58.03 pmol/mL (control < 1.2 ± 0.1 pmol/mL) at BL, 91.27 pmol/mL in male patients (*n* = 12) and 15.30 pmol/mL in female patients (*n* = 14). Both plasma Gb3 and Lyso-Gb3 levels were abnormally high. After the start of ERT, plasma Gb3 and Lyso-Gb3 levels were decreased (Fig. 3c and d), particularly in male patients. In most patients, the levels remained low during ERT period compared to BL.

### Adverse events

The development of anti-agalsidase alfa IgG antibodies was observed in one male patient of the total 36 patients. Although an infusion-associated allergic reaction (urticaria) occurred, it was controllable, and no serious adverse events were observed throughout the observation period.

## Discussion

The JFR-002 is a long-term, prospective, observational study to evaluate efficacy and safety of ERT with agalsidase alfa for 5 years. Treatment with ERT and observation for all patients for the study period were performed in only one institute (Nagoya Central hospital). Also, all clinical tests, including cardiac echogram and laboratory investigations were conducted at one institute, except for measurements of Gb3 and Lyso-Gb3 as special parameters. This situation enabled to perform the evaluation of ERT on various clinical symptoms at heart, kidney and cerebrovascular complications without deviations by using the same evaluative criteria (the same evaluator).

Evidence for both agalsidase alfa and beta has been generated from randomized clinical trials and registry data [5–9, 15–20]. For agalsidase alfa, several reports using the database of Fabry Outcome Survey (FOS) [21], an international, multicenter, collaborative study sponsored by Shire have been reported. Mehta A, et al. [5] conducted a retrospective analysis in 181 patients who were treated with agalsidase alfa for 5 years, and reported that agalsidase alfa improved or maintained cardiac function, renal function, pain score and QoL score. They also documented safety data of agalsidase alfa in 555 patients by a retrospective analysis [5]. Beck M, et al. [6] also conducted a retrospective analysis in 740 patients who were treated with agalsidase alfa. They reported that time to onset of composite events (death and cardiac, renal and cerebrovascular events) was delayed compared to patients not treated with enzyme replacement therapy previously reported (Schffmann et al. [22], Banikazemi et al. [23] and Kampmann et al. [24]).

Germain DP, et al. [7] retrospectively analyzed 52 patients treated with agalsidase beta for 10 years from the Fabry Registry database; an international, multicenter, collaborative study sponsored by Sanofi-Genzyme, and reported that agalsidase beta showed a clinical benefit for controlling progression of both cardiac and renal functions related to Fabry-disease. However, these reports were mostly based on retrospective analyses, indicating it would be difficult to control and remove inter-institutional bias, since it would be an issue to keep accuracy of clinical records, for example difference of medical equipment among institutes. To improve study accuracy, a large scale, randomize, placebo control study would be needed, however, it would be very difficult to conduct such study due to limitation to utilize placebo and of the number of patients with Fabry disease.

There are few reports from well-defined single-center cohorts. Kampmann et al. [8] reported that agalsidase alfa showed clinical effects on cardiac and renal functions, as well as on cardiac structure and symptoms in 45 patients (male 21 and female 24) who were treated with agalsidase alfa for 10 years at a single center in Germany. This study was based on a retrospective analysis of prospectively gathered data.

Therefore, our manuscript can be considered the first report of a study with a single-center, prospective, observation of ERT-treated Fabry patients for 5 years. In addition to this, it is currently one of the few reports on a cohort of ERT treated Japanese Fabry patients.

On cardiac involvement, the electrocardiographic and echocardiographic findings remained stable following ERT intervention, indicating the prevention of cardiac-function decline (Fig. 1a and b).

Plasma BNP level is useful for monitoring several clinical aspects such as heart failure, and for evaluating the effect of ERT during FD. The BNP levels remained within the normal range in 14 patients with grade 1 and gradually decreased in 22 patients with grade 2, which indicated a certain level of improvement, but still stayed within the abnormal range (Fig. 1c and d). These results suggest the efficacy of ERT with agalsidase alfa, and that early intervention with ERT when cardiac or myocardial dysfunction is still mild, may prevent the progression to subsequent cardiac failure and improve cardiac involvement of FD (Fig. 1).

On renal involvement, the eGFR, β2-MG, and NAG levels remained stable following initiation of ERT (Fig. 2a and b), indicating the prevention of decline of renal function. The UP/Cr ratio remained within the normal range in 14 patients classified as stage A1 and generally remained stable in 22 patients classified as stages A2 and A3, indicating that in these patients further progression of renal dysfunction was prevented (Fig. 2c and d). These results suggest the efficacy of ERT with agalsidase alfa, and that early intervention with ERT when renal dysfunction is still mild may prevent the progression of subsequent renal failure and improve renal involvement of FD (Fig. 2).

For the pain score, the scores in 15 patients who had maximum pain of ≥3.0 units at BL, improved from 4.9 units (BL) to 2.1 units (60 M) following ERT intervention (Fig. 3a and b).

For the QoL score, the scores remained steady, from 0.865 (BL) to 0.7982 (60 months) and QoL was steadily maintained throughout treatment with ERT. This is in line with previous findings, which have also shown an improvement in pain and stabilization of QoL in FD patients for both ERT with agalsidase alfa and agalsidase beta [25–28].

The plasma levels of Gb3 and Lyso-Gb3 were markedly decreased in male patients (hemizygotes) after the start of treatment and, in particular, the Lyso-Gb3 level was markedly decreased over the course of treatment, suggesting the efficacy of ERT with agalsidase alfa (Fig. 3c and d). At BL, the plasma levels of Gb3 and Lyso-Gb3 in female patients (heterozygotes) were lower than in their male counterparts, showing no significant difference after ERT intervention; however, there was a gradual downward trend in their levels, suggesting the efficacy of ERT similarly to that in male patients. However, some patients who had cardiac or renal dysfunction before the start of ERT continued the progression of dysfunction in that organ, suggesting the importance of early intervention with ERT adopted before the presence of organ dysfunction.

For the safety of ERT with agalsidase alfa, adverse reactions including laboratory abnormalities were reported in 10 of 12 patients in a clinical study conducted in Japan [29]. The most frequently reported adverse reactions were pyrexia in six patients, chills, and malaise in four patients each, and pain in extremity, feeling hot, CPK increase, and dyspnea in two patients each. No serious adverse reactions were reported [29]. In a clinical trial conducted overseas, adverse reactions were reported in 41 out of 65 patients (63%). The most frequently reported adverse reactions were flushing in 14 patients (22%), chills in 12 patients (18%), pyrexia in 9 patients (14%), nausea in 8 patients (12%), and headache in 7 patients (11%). However, no serious adverse reactions were reported [29]. In this study, one male patient out of 36 patients developed anti-agalsidase alfa IgG antibodies, and experienced an infusion-associated allergic reaction (urticaria). Since the urticaria was noted upon treatment with agalsidase alfa, the administration time of infusion was changed from 40 to 60 min and the patient orally took D-chlorpheniramine maleate and betamethasone before the start of infusion. After these measures were taken, infusion-associated urticaria did not occur. This patient has continued to receive ERT periodically until recently. He has tested positive for agalsidase alfa IgG antibodies, but infusion related reactions such as urticaria have no longer been observed.

Throughout the follow-up period, no serious adverse events were observed. Similar to reports obtained from within and outside Japan [21, 30], the results of this study confirm that ERT with agalsidase alfa 0.2 mg/kg every 2 weeks is well tolerated, and that immunogenicity of agalsidase alfa was low.

## Conclusion

In the JFR-002, effectiveness and safety of ERT were examined in 36 patients with FD who received ERT with agalsidase alfa at the approved dose of 0.2 mg/kg every 2 weeks. Although the assessment required careful analyses due to the limited number of patients, our study suggests that agalsidase alfa is effective in maintaining multiple organ function in FD patients, and that the incidence of infusion reactions related to the treatment with agalsidase alfa is low, indicating the good tolerability of this ERT.

## Abbreviations

Alb: Albumin
ALP: Alkaline phosphatase
ALT: Alanine aminotransferase
AST: Aspartate transaminase
BL: Base line
BNP: Brain natriuretic peptide
BP: Blood pressure
BPI: Brief pain inventory
BUN: Blood urea nitrogen
CK: Creatinine kinase
Cl: Chlorine
Cre: Creatinine
CRP: C-reactive protein
ECG: Electrocardiography
EF: Left ventricular ejection fraction
eGFR: Estimated glomerular filtration rate
EQ-5D: Euro QoL- 5 dimension
EQ-VAS: EQ-visual analogue scale
ERT: Enzyme-replacement-therapy
FD: Fabry disease
FT3: Free triiodothyronine
FT4: Free thyroxine
Gb3: Globotriaosylceramide
Hb: Hemoglobin
HDL: High-density lipoprotein
hs-Trop I: High-sensitivity troponin I
Ht: Hematocrit
IVSd: Interventricular septal thickness in diastole
K: Potassium
LDH: Lactase dehydrogenase
LDL: Low-density lipoprotein
LVMI: Left ventricular mass index
LVpwd: Left ventricular posterior wall thickness in diastole
Lyso-Gb3: Globotriaosylsphingosine
Na: Sodium
NAG: N-acetyl-beta-D-glucosaminidase
Plt: Platelet
QoL: Quality of life
RBC: Red blood cell count
T-Bil: Total bilirubin
T-Cho: Total cholesterol
TG: Triglyceride
TP: Total protein
TSH: Thyroid-stimulating hormone
UA: Uric acid
UCG: Ultrasonic cardiography
UP/Cr ratio: Urine protein/creatinine ratio
WBC: White blood cell
α-Gal: Alpha-galactosidase A
β2-MG: Beta2-microglobulin
γ-GTP: Gamma-glutamyl transferase
